# Proportions of Maxillary Anterior Teeth Relative to Each Other and to Golden Standard in Tabriz Dental Faculty Students

**DOI:** 10.5681/joddd.2010.021

**Published:** 2010-09-16

**Authors:** Fereydoun Parnia, Ali Hafezeqoran, Farhang Mahboub, Elnaz Moslehifard, Rodabeh Koodaryan, Rosa Moteyagheni, Fariba Saleh Saber

**Affiliations:** ^1^ Assistant Professor, Department of Prosthodontics, Faculty of Dentistry, Tabriz University of Medical Sciences, Tabriz, Iran; ^2^ Post-graduate Student, Department of Prosthodontics, Faculty of Dentistry, Tabriz University of Medical Sciences, Tabriz, Iran

**Keywords:** Anterior teeth, esthetics, golden proportion, maxilla

## Abstract

**Background and aims:**

Various methods are used to measure the size and form of the teeth, including the golden pro-portion, and the width-to-length ratio of central teeth, referred to as the golden standard. The aim of this study was to eval-uate the occurrence of golden standard values and golden proportion in the anterior teeth.

**Materials and methods:**

Photographs of 100 dentistry students (50 males and 50 females) were taken under standard conditions. The visible widths and lengths of maxillary right and left incisors were calculated and the ratios were compared with golden standard. Data was analyzed using SPSS 14 software.

**Results:**

Review of the results of the means showed statistically significant differences between the width ratio of right lateral teeth to the central teeth width with golden proportion (P<0.001). Likewise, the difference was significant for the left side, too (P<0.001). Test results of mean differences showed that the mean difference between proportion of right laterals to centrals with golden proportion was significant (P<0.001). The difference was significant for the left side, too (P<0.001). As a result, there is no golden proportion among maxillary incisors. The review of results of mean differences for single samples showed that the mean differences between the proportion of width-to-length of left and right central teeth was statistically significant by golden standard (P<0.001). Therefore, considering the width-to-length proportion of maxillary central teeth, no golden standard exists.

**Conclusion:**

In the evaluation of the width-to-width and width-to-length proportions of maxillary incisors no golden proportions and standards were detected, respectively.

## Introduction

In treating patients with missing maxillary anterior teeth, dental practitioners must determine tooth size and shape to achieve an optimal aesthetic result.^1^ If the size and shape of a replaced tooth are not in harmony with patients’ face and other teeth, psychological and social problems might arise.^2^

Several authors have presented guidelines regarding anterior aesthetics in order to achieve excellent aesthetics. One of the most important guidelines is golden standard value. According to this standard, the optimal width-to-length proportion of maxillary central incisor varies between 66% and 85%.^2 , 3^ Lombardi^5^ proposed that dental and facial aesthetics are optimized if central incisor-to-lateral incisor width and lateral incisor-to-canine width are repeated in proportion when the patient is viewed from the front. This proportion was called golden proportion and is approximately 1.618 to 1. In this manner the visible width of maxillary lateral incisor is 62% of central incisor and the visible width of canine is 62% of lateral incisor.^4^

Wolfart et al^2^ evaluated the subjective judgment of patients about their own dental appearance and correlated the results with objective measurements of their dentitions concerning the appearance of maxillary incisors. Objective measurements were evaluated with regard to four parameters, including length of maxillary central incisors, their length exposed during laughing, width-to-length ratio of central incisors and the proportion between the width of the lateral and central incisors. The results of this study showed that there were no differences in the objective measurements between genders but the degree of satisfaction concerning the appearance of maxillary incisors according to golden standard values was higher for men than for women; therefore, it can be concluded that women might be more influenced by emotions and that they have more critical judgment concerning beauty criteria.

Hasanreisoglu et al^6^ evaluated the dimensions of anterior teeth, the occurrence of golden proportion and several dental and facial measurements using full-face and anterior teeth images and gypsum casts of the maxillary arches of 100 Turkish dental students. The results showed that the dimensions of the central incisors and canines varied by gender; the existence of golden proportion was not substantiated; and proportional relationships were observed in women between the bizygomatic width and the width of the central incisor, and the inter-canine distance and the inter-alar width.

Wolfart et al^3^ evaluated the attractiveness of standardized changes in incisor proportions using computer-manipulated photographs in another study. Standardized changes were made in the width-to-length ratios of central incisors and in tooth-to-tooth proportions between the width of lateral and central incisors. The width-to-length ratios were assessed as most attractive within the range of 75-85% and the tooth-to-tooth proportions showed best results concerning attractive appearance within a range of 50-74%.

The aim of this study was to evaluate the occurrence of golden standard values and golden proportion in the anterior teeth in a population of Iranian students.

## Materials and methods

For the purpose of this descriptive study, data was collected using photographic technique. Sample size was determined to be 97 individuals (α=0.05, β = 0.5 and d=0.098). Written requests were sent to each dental student (Tabriz Faculty of Dentistry) to participate in this study. Participants (50 females and 50 males) were selected according to the following criteria:

1. Complete upper and lower anterior teeth
2. No periodontal disease
3. No spacing and crowding in anterior maxillary teeth
4. No history of orthodontic treatments
5. No intruded, extruded or rotated teeth in the anterior region

Using a digital camera (Canon, Song-Model F707, Japan), a frontal photograph was taken from each individual with an esthetic smile. The upper lip was retracted in all the photographs to clearly display maxillary anterior teeth as well as their respective gingiva. Lighting and distance were kept constant. Using the cephalostat of a panoramic radiography device, photographs were taken with the head in upright position, Frankfort plane parallel with the horizon, and the center of the lens of the camera coincident with the participant’s midline. The actual sizes of the teeth were calculated with a ruler by taking into account the magnification of the camera in the working field.

All the photographs were evaluated by Photoshop software. Since angulations of a tooth make its visible width different from its actual width, we measured the visible width of the teeth. The length and width of clinical crowns of central maxillary incisors were also measured. Visible width of upper anterior teeth and the ratio of width-to-length of each tooth was compared with golden proportion (Figure 1).

**Figure:**
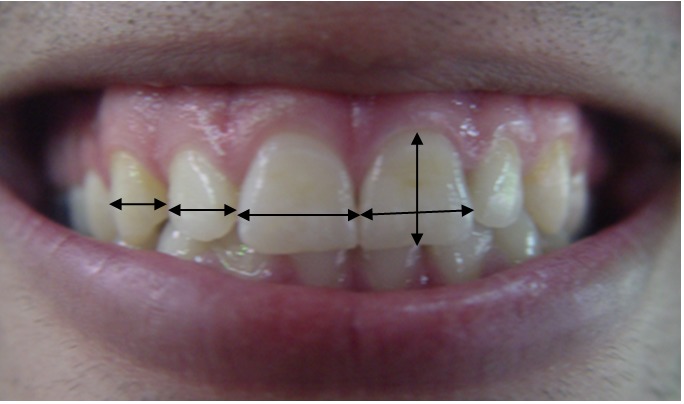
Figure 1.

Figure 1. Visible width of upper anterior teeth and width-to-length ratios of central incisors were measured by Photoshop software.

Descriptive statistics and one-sample t-test were used to analyze data with SPSS/Win 14 software. A 1% level of significance was chosen.

## Results

In this research 50 females and 50 males, with a age range of 20-27 years, were assessed. The average age was 23.57 ± 2.09 (mean ± SD). More than half of the subjects were more than 24 years old. The length and width of maxillary anterior teeth are summarized in Table 1.

**Table 1:** Length and width of maxillary anterior teeth in millimeters

Tooth	Mean ± SD	Minimum	Maximum
Length of right central incisor	9.19 ± 1.65	6	12.2
Length of left central incisor	9.22 ± 1.68	6	12.4
Width of right central incisor	7.70 ± 1.57	4.8	10.4
Width of right lateral incisor	5.05 ± 0.87	3	7.4
Width of right canine	3.29 ± 0.56	2.2	4.4
Width of left central incisor	7.62 ± 1.51	4.8	10.4
Width of left lateral incisor	4.81 ± 0.87	3	7.4
Width of left canine	3.23 ± 0.53	2	4.4

According to the results of one-sample t-test, there was a statistically significant difference between the proportion of the right lateral incisor width and the right central incisor width based on golden proportion (P<0.001). For the left side of the jaw, the difference was also significant (P<0.001).

This difference between the assumed proportion of the right segment and golden proportion was statistically significant. The same situation was observed on the left side. Therefore, considering the visible width of the upper incisor teeth, no golden proportion existed.

In addition, the proportion of width-to-length of left and right central incisors in comparison with golden standard was statistically different, too (P<0.05).

**Table 2:** Proportions of maxillary anterior teeth width relative to each other and significance level of one-sample t-test for comparison with golden proportion

Tooth	Mean	SD	P value
Width of right lateral incisor to width of right central incisor	0.66	0.069	0.0001
Width of right canine to width of right lateral incisor	0.66	0.13	0.0001
Width of left lateral incisor to width of left central incisor	0.63	0.08	0.0001
Width of left canine to width of left lateral incisor	0.68	0.13	0.0001

**Table 3:** Width-to-length ratios of maxillary central incisors and significance level of one-sample t-test for comparison with golden proportion

Tooth	Mean	SD	P value
Right central incisor	0.83	0.055	0.0001
Left central incisor	0.82	0.048	0.0001

Again in assessment of the width-to-length proportions of maxillary central teeth, no golden standard was observed.

## Discussion

The Iranian population is relatively heterogeneous with many dental and facial variations as a result of its racial characteristics.^7^ Dental aesthetics is dependent on many different factors and interrelationships between aesthetically relevant factors. Therefore, information regarding golden proportions may prove useful to clinicians in the esthetic zone but racial differences should be taken into account. The size and morphology of maxillary central incisors are the key determinants in esthetically accepted cases. In traditional methods extracted teeth were used for measuring tooth sizes, but at present photographs and casts are used for this purpose instead of extracted teeth.^6^ This study evaluated visible widths of maxillary centrals, laterals, canines and the lengths of central teeth. The measurements were compared with golden proportion and golden standard.

The crown width-to-height ratio has been accepted as the most stable reference by authors.^6^ In the present study, in the right maxillary central incisors, width-to-height ratio mean was 83% ± 0.05%, with a minimum of 72% and a maximum of 96%. On the left side, width-to-height ratio of centrals was 82% ± 0.04%, with a minimum of 71% and a maximum of 94%. The results are different in comparison to the mean 80% noted in the dental literature as golden standard. In Hasanreisoglu investigation on Turkish population, the width-to-height ratios of maxillary anterior teeth in both genders were 76-86%. It seems that maxillary anterior teeth in our study were narrower compared to the teeth in Hasanreisoglu study, which might be attributed to differences in racial characteristics. Our results are similar to the results of a study carried out by Wolfart,^3^ who reported a width-to-length proportion of 82%.

In general, this study and several other surveys have estimated that there is no golden standard in the nature but the optimal width-to-height ratio of upper central incisors is dominating the aesthetic factor and can be used in restoring maxillary upper teeth.

We studied relative proportions of central and lateral incisors and canines according to the golden proportion of 1.618 and found no relationships. In the research on maxillary anterior teeth in the University of Ankara, they achieved the same result but they emphasized that they selected their participants randomly and not on the basis of esthetic properties. Rather than focusing on the 62% proportion, Rosenstiel et al^1^ recommended using a ratio of 70%.

Many studies have reported that rather than concentrating on a single ratio, such as the golden proportion, other ratios reflecting harmony among tooth lengths should be considered when striving to produce a satisfactory appearance.^1 , 7 , 8^

## Conclusion

From the present study, the following conclusions were drawn:

1. There were no statistically significant differences between proportions of width-to-height of central incisors and golden standard.
2. We achieved 0.76 to 0.86 for the proportions of width-to-height in central incisors.
3. We could not find any proportions of width of the maxillary central incisors, lateral incisors and canines from the frontal view.
